# Open-Label Fosmetpantotenate, a Phosphopantothenate Replacement Therapy in a Single Patient with Atypical PKAN

**DOI:** 10.1155/2017/3247034

**Published:** 2017-04-16

**Authors:** Yiolanda-Panayiota Christou, George A. Tanteles, Elena Kkolou, Annita Ormiston, Kostas Konstantopoulos, Maria Beconi, Randall D. Marshall, Horacio Plotkin, Kleopas A. Kleopa

**Affiliations:** ^1^Neurology Clinics, The Cyprus Institute of Neurology and Genetics, Nicosia, Cyprus; ^2^Clinical Genetics Clinic, The Cyprus Institute of Neurology and Genetics, Nicosia, Cyprus; ^3^European University Cyprus, Nicosia, Cyprus; ^4^Retrophin Inc., New York, NY, USA

## Abstract

*Objective*. Pantothenate kinase-associated neurodegeneration (PKAN) is an autosomal recessive disorder with variable onset, rate of progression, and phenotypic expression. Later-onset, more slowly progressive PKAN often presents with neuropsychiatric as well as motor manifestations that include speech difficulties, progressive dystonia, rigidity, and parkinsonism. PKAN is caused by biallelic* PANK2* mutations, a gene that encodes pantothenate kinase 2, a regulatory enzyme in coenzyme A biosynthesis. Current therapeutic strategies rely on symptomatic relief. We describe the treatment of the first, later-onset PKAN patient with oral fosmetpantotenate (previously known as RE-024), a novel replacement therapy developed to bypass the enzymatic defect.* Methods*. This was an open-label, uncontrolled, 12-month treatment with fosmetpantotenate of a single patient with a later-onset, moderately severe, and slowly progressive form of PKAN.* Results*. The patient showed improvement in all clinical parameters including the Unified Parkinson's Disease Rating Scale (UPDRS), Barry-Albright Dystonia Scale, the EuroQol five-dimensional three-level (EQ-5D-3L) scale, timed 25-foot walk test, and electroglottographic speech analysis. Fosmetpantotenate was well-tolerated with only transient liver enzyme elevation which normalized after dose reduction and did not recur after subsequent dose increases.* Conclusions*. Fosmetpantotenate showed promising results in a single PKAN patient and should be further studied in controlled trials.

## 1. Introduction

Pantothenate kinase-associated neurodegeneration (PKAN) is the most common form of neurodegeneration with brain iron accumulation (NBIA). It is an autosomal recessive disorder resulting from biallelic mutations in the* PANK2* gene on chromosome 20p13 [1, 2] for which there is only symptomatic treatment. PKAN has traditionally been divided into a classic, earlier-onset form and an atypical, later-onset form which typically shows slower progression [2]. The presence however of patients with overlapping features between the two forms is increasingly becoming evident. The majority of PKAN patients are diagnosed in the first 10 years of life with affected children usually losing the ability to walk within 10 to 15 years after disease onset [3]. Clinical manifestations of PKAN have highly variable age of onset and rate of progression and include developmental delay, dystonia sometimes causing intractable pain, choreoathetosis, dysarthria, spasticity, rigidity, parkinsonism with gait freezing and bradykinesia, retinal degeneration, and dysphagia [3, 4]. Age of onset and rate of progression are variable, with some patients progressing rapidly to death within a few years, whereas others may live into later adulthood with more slowly progressive symptoms [3–6]. In most patients, brain MRI reveals the typical “eye-of-the-tiger” sign, resulting from excessive brain iron deposition in the basal ganglia. MRI changes may precede clinical manifestations [2, 3].

The normal product of* PANK2* is a pantothenate kinase which is essential in coenzyme A (CoA) biosynthesis and catalyzes the phosphorylation of pantothenate (vitamin B5) to phosphopantothenic acid (PPA).* PANK2* mutations can generally be divided into null or missense and only a few are recurring. Missense mutations (majority of identified variants) can lead to either early or late onset PKAN forms. Generally, no clear genotype-phenotype correlation has been observed except in the case of homozygous individuals for null alleles who usually present with classic disease [7].

*PANK2* mutations are thought to result in deficiency (complete or partial) of pantothenate kinase 2 and accumulation of cysteine-containing cytotoxic substrates [9]. Pantothenate kinase 2 deficiency is also predicted to deplete CoA leading to multiple downstream consequences including defective membrane biosynthesis depending on specific tissue demand [10]. It has been postulated that, in PKAN patients, PPA deficiency leads to decreased CoA levels [5]. A phosphopantothenate replacement therapy to bypass the genetic deficiency in the Pank1−/− mouse model recently showed that administration of selected candidate compounds corrected their deficiency in hepatic CoA [11]. The results provided strong support for PanK as a master regulator of intracellular CoA and illustrated the feasibility of employing PanK bypass therapy to restore CoA levels in genetically deficient mice.

**Figure 1:**
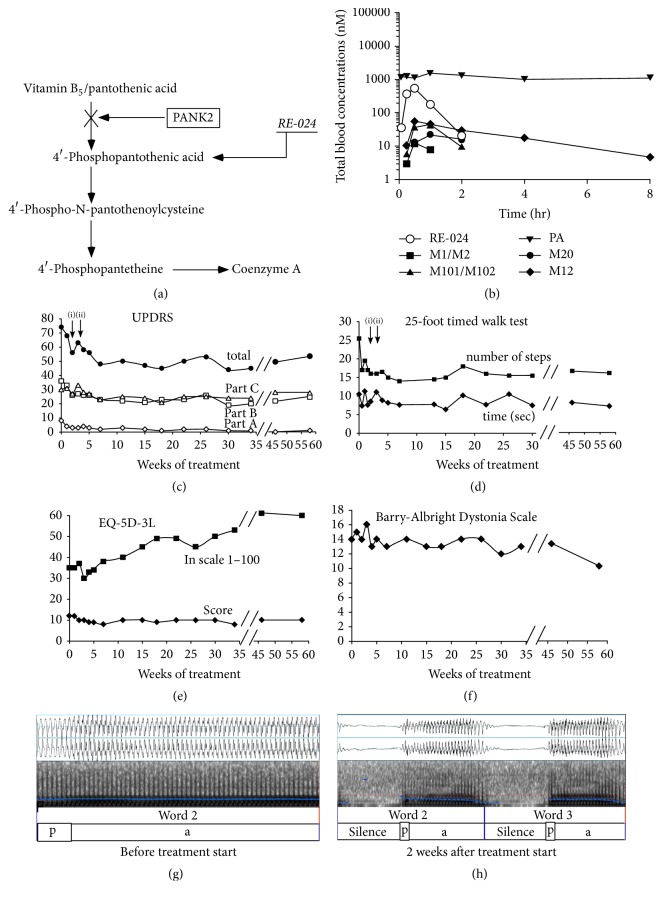
*Mode of Action, Pharmacokinetics, and Clinical Effects of Fosmetpantotenate*. (a) Diagram showing the metabolic pathway from pantothenic acid to coenzyme A with the pantothenate kinase defect found in PKAN patients and the bypassing effect of fosmetpantotenate. (b) Whole blood concentrations of fosmetpantotenate and metabolites over time following a 1 mg/kg oral dose on Day 7 of treatment. (c–f) Clinical evaluations before and after 58 weeks of treatment with fosmetpantotenate. (c) Unified Parkinson's Disease Rating Scale (UPDRS) including parts C and D and total score. (d) Timed 25-foot gait test including number of steps and time to complete (arrows in diagrams (c) and (d) indicate (i) interruption of treatment; (ii) restarting treatment at half dose). (e) Quality of life scale (EQ-5D-3L). (f) Barry-Albright Dystonia Scale. (g-h) Representative illustrations of electroglottographic analysis [8] before (g) and two weeks after (h) starting treatment showing individual /pa/ segmentation. Before treatment there is an apparent trend to prolong the vowel while after medication there is a significant improvement in the production of /pa/.

Fosmetpantotenate (previously known as RE-024) was developed by Retrophin, Inc. to bypass this biochemical defect for the treatment of PKAN. It is a novel small molecule precursor of PPA designed to release PPA intracellularly, leading to restoration of CoA levels (Figure 1(a) and Supplementary Figure 1, in Supplementary Material available online at https://doi.org/10.1155/2017/3247034). It has been reported that 4′-phosphopantothenic acid is not permeable to cell membranes, and thus systemic administration of PPA, the enzymatic product of PanK2, to PKAN patients will not be effective. In contrast, fosmetpantotenate has been shown to significantly increase intracellular CoA levels and increase tubulin acetylation in vitro in neuroblastoma cells that have been silenced for PanK2 expression [12, 13], presumably via access to mitochondrial PanK2. When dosed orally in nonhuman primates, microdialysis sampling detected fosmetpantotenate in the brain at levels consistent with meaningful conversion to PPA [13], suggesting similar BBB in humans.

We have in the past reported on a PKAN family in which a novel biallelic missense variant in exon 2 of the* PANK2* gene [c.695A>G(p.Asp232Gly)] was identified [14]. Here, we present the results of the first oral replacement therapy with fosmetpantotenate in a single patient from this family with onset of PKAN symptoms in his early 20s.

## 2. Case Description and Evaluation Methods

The oral replacement therapy results presented here involve a Cypriot patient affected with later-onset PKAN who was homozygous for the novel c.695A>G (p.Asp232Gly) missense mutation in exon 2 of the* PANK2* gene [14]. He initially presented with gait instability at the age of 22 years and subsequently developed progressive trunk and limb rigidity, more prominent on the right lower limb, impairing his ability to walk unaided, with frequent falls (Supplementary Video). By the age of 27 he presented to a neurology clinic with dysarthria, poor concentration (mild bradyphrenia), palilalia, bradykinesia in both hands, and marked axial rigidity. His gait was characterized by leg spasticity, toe walking, and moderate instability. MRI revealed the “eye-of-the-tiger” sign. He was unable to walk without support having a tendency to fall backwards. He showed a continuous progression and became wheelchair bound for most of the time by the age of 34. There was also worsening of dysarthria, dysphonia with palilalia, and dysphagia along with mild concentration difficulties and obsessive-compulsive behavior. Treatment with fosmetpantotenate was initiated at the age of 35.

After receiving a Named Patient approval for fosmetpantotenate administration from Government Authorities according to national law (Ph.S.5.21.2.1.6), informed consent was obtained, and fosmetpantotenate treatment was initiated with a starting dose of 0.1 mg/kg twice daily orally and it gradually increased over one week to 240 mg (3 mg/kg/day), divided equally as three times daily.

A baseline evaluation was performed with weekly follow-up visits for the first two months and every month thereafter. Clinical evaluation included the Unified Parkinson's Disease Rating Scale (UPDRS/I,II,III) [15], the Barry-Albright Dystonia (BAD) Scale, the EuroQol five-dimensional three-level (EQ-,5D-3L) scale, and the timed 25-foot walk test.

Electroglottographic analysis was performed noninvasively [8] before and two weeks after starting treatment. Variables used for voice analysis included average fundamental frequency and jitter as well as the average time in milliseconds for the production of speech diadochokinesis. The patient was asked to perform a rapid repetition of consonant-vowel pairs sequences on a single breath such as /p∧/, /t∧/, and /k∧/ defined as Alternating Motion Rates (AMRs) and the average time in milliseconds for the production of consonants /p, t, k/ was registered.

Whole blood samples were collected for pharmacokinetic analysis on treatment Day 7 before dose and at 0.0833, 0.25, 0.5, 1, 2, 4, and 8 hours after dose. Samples were acidified immediately upon collection, treated with an enzyme inhibitor, and stored frozen until analysis. Fosmetpantotenate and metabolites were quantified under GLP conditions, by liquid chromatography tandem mass spectrometry (LC-MS/MS), using methods developed and GLP-validated by Nextcea (Worcester, MA) (Figure 1(b)). Metabolites quantified included fosmetpantotenate derivatives containing the intact PPA moiety, PA, and PPA.

## 3. Results

### 3.1. Dosing, Tolerance, and Safety

Fosmetpantotenate was well-tolerated with only mild nausea after taking the medication the first 2 days, which resolved without treatment or dose adjustment. At the end of week 2, transaminase increases were noted (2-3-fold) and treatment was stopped on Day 14. Transaminases rapidly decreased, and treatment was restarted on Day 22 at 120 mg daily (1.5 mg/Kg/day; half dose). Dosing was gradually increased to 150 mg daily (1.9 mg/kg/day) on Week 15 and gradually to 210 mg daily (2.64 mg/kg/day, divided tid) by Week 40 without any further adverse effects or clinically significant laboratory or electrocardiogram abnormalities through 12 months of treatment.

### 3.2. Pharmacokinetic Studies

Following the 1 mg/kg dose, fosmetpantotenate showed a short half-life (*t*_1/2_ = 0.3 h) and was detectable for 2 hours. Fosmetpantotenate metabolite concentrations were at least 10 times lower than those of fosmetpantotenate but detectable for a longer period of time. The M12 metabolite had the highest *C*_max_ and was detectable in circulation through 8 hours after dose, with a *T*_1/2_ of 2.1 hours (Figure 1(b)).

### 3.3. Clinical Evaluation

Clinical benefit from fosmetpantotenate was observed based on multiple outcome measures, addressing the multiple manifestations of the disease, including continuous improvement of the patient's ability to walk in the first two months of treatment and stabilization thereafter during one year of follow-up. At baseline, the patient required another person's assistance when walking. After treatment, on the timed 25-foot walk test he was able to walk faster with longer steps and for a few meters without assistance (Figure 1(d) and Supplementary Video). Dystonia, parkinsonism, rigidity, and bradykinesia together with overall quality of life (QoL) improved as noted on the UPDRS, BAD, and EQ-5D-3L scales (Figures 1(c)–1(f)). During the one week of dose interruption due to elevated transaminases all clinical scales deteriorated but improved again upon restarting treatment.

### 3.4. Electroglottographic Analysis

In sustained phonation of the /a/ sound, the average fundamental frequencies of voice and the jitter (cycle-to-cycle variation in fundamental frequency) were lowered after medication (123.77 Hz; jitter 0.50%) as compared to previously (139.97 Hz; jitter 0.64%) (normal for males: around 125 Hz; jitter < 1) [8]. Acoustic analysis of the AMRs showed similar trends in the mean production of the sounds /p, t, k/ (/p/: 0.05 msec before and 0.01 msec after treatment; /t/: 0.02 msec before and 0.01 msec after; /k/: 0.07 msec before and 0.01 msec after treatment) (Figures 1(g) and 1(h)).

### 3.5. Brain MRI

The baseline brain MRI scan was characterized by bilateral peripheral hypointensity in the globi pallidi with a central focus of gliosis, the typical “eye-of-the-tiger” sign [14]. Follow-up MRI performed after eight months of treatment showed no significant change.

## 4. Discussion

Despite rapid developments in understanding the genetics, pathophysiology, and clinical presentation of PKAN in the last decade, only symptomatic relief therapies are available [4, 16]. Bilateral deep brain stimulation of the globus pallidus was shown to partially improve dystonia severity in a multicenter retrospective study of 23 PKAN patients [17] and in a cohort of 7 children [18]. Repetitive transcranial magnetic stimulation 1-Hz of premotor cortex was experimentally used in a single patient and resulted in mild transient improvement [19]. A phase II pilot open trial of the iron-chelator deferiprone in 10 PKAN patients for a period of six months showed reduction in pallidal iron content but with no significant clinical improvement [20], whereas a more recent four-year study of six patients with NBIA, including five with PKAN, resulted in clinical stabilization correlating with radiological improvement [21].

In our patient, oral treatment with fosmetpantotenate proved to be safe and well-tolerated with only transient nausea and mild transaminase elevation, which resolved after dosage adjustment and has not recurred with gradually increasing doses. However, safety and tolerability of this treatment remain to be confirmed in larger numbers of patients.

In 2015, Tomić et al. [22] evaluated the clinical features and disease course of nine molecularly confirmed patients with atypical PKAN. The majority of patients reached seven significant clinical milestones in the first 4.6 years during the course of the disease. A long-lasting, relatively stable period of slower progression followed. This period was mostly complicated by skeletal deformities (developing after 7.0 ± 2.8 years) [22]. The study did not show any periods of clear clinical improvement being part of the natural course of the disease.

In our patient clinical benefit from fosmetpantotenate was observed based on multiple outcome measures, addressing the multiple manifestations of this disease, including dystonia, parkinsonism, and speech dysfunction [5]. Our study showed that, following a single oral administration, fosmetpantotenate enters the blood stream. Metabolites of fosmetpantotenate were detectable, but with no change in levels of pantothenic acid (PA), suggesting that PA from fosmetpantotenate is diluted by endogenous pantothenic acid pools to undetectable levels. Its half-life suggests that more than one daily dose may be needed for adequate coverage. In this patient, fosmetpantotenate was administered three times daily. Although it seems unlikely that the drug will reverse neurodegeneration, it may improve function of surviving cells, thereby preventing disease progression. This also underscores the importance of early treatment in future studies.

Since this is a single case we cannot exclude the possibility of a placebo effect or the effect of study participation, especially on the QoL scores. Furthermore, disease progression in this patient, though relentless, was at the slower end of the spectrum, and longer observation periods of over a year may be needed to confirm a lasting effect. Controlled studies will be required to demonstrate a real benefit from this treatment.

*In conclusion*, this open-label, uncontrolled, 12-month treatment of a single atypical PKAN patient with fosmetpantotenate was associated with transient nausea and mild liver enzyme elevations which resolved with treatment cessation and did not recur upon rechallenge. Most importantly, the treatment was associated with stabilization and persistent improvement of the patient's symptoms. Although symptom stabilization has clearly been reported in the literature, the improvement of symptoms observed in our patient is clinically meaningful and represents something not reported to be part of the natural course of the disease. Controlled studies should evaluate the therapeutic potential of fosmetpantotenate in patients with PKAN.

## Supplementary Material

Legend to Supplementary video: Timed 25 foot gait test before starting treatment (first part) and 2 weeks afterwards (second part). In the initial test the patient is walking behind his mother holding onto her shoulders with both hands for support. He starts with small steps leaning greatly forwards and placing weight on toes, then gradually takes bigger steps with foot placement in contact with the floor. In the second test he walks behind his father without any support and with faster steps. He maintains good upright posture achieving good foot placement with the sole of the foot in contact with the floor with some dystonia still present, more so in the right foot.
Supplementary Figure 1: postulated mechanism and chemical structure of fosmetpantotenate.
