# Supplementary material for: Open-Label Fosmetpantotenate, a Phosphopantothenate Replacement Therapy in a Single Patient with Atypical PKAN
Source: Case Rep Neurol Med. 2017 Apr 16;2017:3247034. doi: 10.1155/2017/3247034 (PMC5439260; doi:10.1155/2017/3247034)
Supplement: Supplementary file 1 — Legend to Supplementary video: Timed 25 foot gait test before starting treatment (first part) and 2 weeks afterwards (second part). In the initial test the patient is walking behind his mother holding onto her shoulders with both hands for support. He starts with small steps leaning greatly forwards and placing weight on toes, then gradually takes bigger steps with foot placement in contact with the floor. In the second test he walks behind his father without any support and with faster steps. He maintains good upright posture achieving good foot placement with the sole of the foot in contact with the floor with some dystonia still present, more so in the right foot. Supplementary Figure 1: postulated mechanism and chemical structure of fosmetpantotenate. [file 3247034.f1.zip › New Microsoft Word Document.docx]

**SUPPLEMENTARY MATERIAL**

**Legend to Supplementary video:** Timed 25 foot gait test before starting treatment (first part) and 2 weeks afterwards (second part). In the **initial test** the patient is walking behind his mother holding onto her shoulders with both hands for support. He starts with small steps leaning greatly forwards and placing weight on toes, then gradually takes bigger steps with foot placement in contact with the floor. In the **second test** he walks behind his father without any support and with faster steps. He maintains good upright posture achieving good foot placement with the sole of the foot in contact with the floor with some dystonia still present, more so in the right foot.

**Supplementary Figure 1:** postulated mechanism and chemical structure of fosmetpantotenate.
